# Potential roles of colostrum microbiota in shaping calf gut microbiota and colostrum metabolites

**DOI:** 10.3168/jdsc.2025-0883

**Published:** 2026-01-16

**Authors:** Jalyn Hawkins, Shelby Carpenter, Himani Joshi, Chuan-Yu Hsu, Caleb Lemley, Peixin Fan

**Affiliations:** 1 Department of Animal and Dairy Sciences, Mississippi State University, Mississippi State, MS 39762; 2 Institute for Genomics, Biocomputing & Biotechnology, Mississippi State University, Mississippi State, MS 39762

## Abstract

Colostrum provides vital nutrients and antibodies that are crucial for calf health and survival. It also contains microbes that may be vertically transmitted to calves and influence early gut microbiota development. These microbes in colostrum may also biologically function to produce unique metabolites that affect colostrum quality and calf growth. However, these colostrum components remain largely unexplored. The aim of this study was to identify colostrum-derived microbes capable of colonizing and persisting in the calf gut and to characterize colostrum metabolomics in relation to the colostrum microbiota. Colostrum samples were collected from 25 Holstein heifers, and fecal samples were collected from their individually housed Holstein-Angus crossbred offspring on d 4, 7, 14, and 30 after birth. Colostrum and fecal microbiota were analyzed using full-length 16S rRNA gene amplicon sequencing, whereas the untargeted metabolomics was performed using ultra-performance liquid chromatography MS. We identified 20% prevalent colostrum bacteria (15 species) were consistently detected in calf fecal samples across all time points, indicating their potential to colonize and persist in the early gut, although the relative abundance of these species in calf feces gradually decreased from d 4 to 30. Colostrum samples were classified into 3 distinct clusters based on the dominant species: *Streptococcus thermophilus*, *Lactococcus lactis*, and *Comamonas testosteroni*. Three colostrum samples from each cluster were selected as a focal group for the untargeted metabolomics analysis. We identified a total of 405 metabolites present in the colostrum samples. No significant differences in metabolomic profiles were observed among the 3 microbial clusters, indicating that colostrum microbiota were not the main drivers of metabolomic dynamics. However, 54 strong positive correlations were detected between bacterial species and metabolites, particularly between colostrum-calf feces shared species and microbial-derived metabolites. For example, 4-methylphenol was positively associated with *Bacteroides fragilis*, the most abundant bacterial species in calf feces on d 4. In addition, *Streptococcus uberis*, a pathogen associated with mastitis, exhibited the greatest number of strong negative correlations with metabolites. In conclusion, this study identified specific colostrum bacterial species with the potential to transmit and persist in the calf gut microbiota and to contribute to microbial metabolite production. Further research is warranted to evaluate the roles of these persistent microbes and their metabolites in shaping colostrum quality, calf growth, and health outcomes.

## Highlights

• Approximately 20% of colostrum bacteria persisted in the calf gut during the first month of life.
• Colostrum metabolomic dynamics were not mainly driven by the colostrum microbial profile.
• Strong associations were detected between colostrum bacteria and metabolites.
• Streptococcus uberis exhibited the most negative associations with metabolites among colostrum bacteria.
• Future work should validate the effect of microbial-associated colostrum metabolites on calves.

## Graphical Abstract

**Figure:**
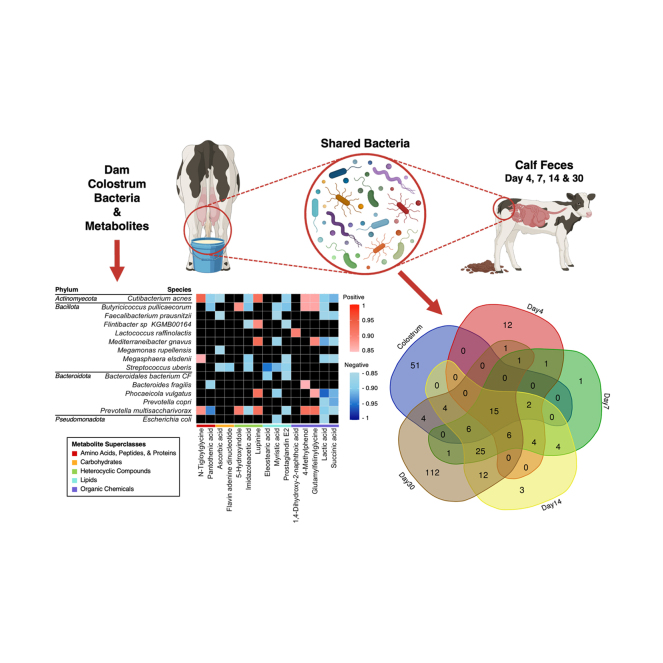
**Summary:** Colostrum is the first and essential meal in a calf's life; however, research investigating the microbial and metabolic profiles of colostrum in relation to calf gut microbiota remains limited. In this study, 15 bacterial species identified in colostrum were persistently detected in calf feces throughout the first month of life, indicating their potential to colonize the gut and influence the microbiota development of calves. Based on bacterial composition, colostrum samples were classified into 3 clusters, though no significant differences were observed in the metabolomic profiles between the clusters. Nevertheless, multiple associations were detected between colostrum bacteria and metabolites, including 4 of the 15 shared species that were positively correlated with microbial-derived metabolites. These microbial-metabolite interactions warrant further investigation to clarify their potential roles in colostrum quality and calf health. Created in BioRender.

Colostrum is the initial diet for newborn calves, known for providing essential nutrients, growth factors, and immunoglobulins critical for their survival (Osaka et al., 2014). Additionally, colostrum contains microbes, which are dominated by the bacterial phyla *Bacillota* (e.g., *Staphylococcus*, *Streptococcus*, and *Lactobacillus* spp.), *Bacteroidota* (e.g., *Prevotella* and *Bacteroides* spp.), *Pseudomonadota* (e.g., *Pseudomonas* and *Escherichia* spp*.*), and *Actinomycetota* (e.g., *Corynebacterium* spp.; Lima et al., 2017; Vasquez et al., 2022). The bacterial composition of colostrum can vary due to dam parity, as well as mammary infections such as mastitis, resulting in greater abundances of potentially pathogenic phyla *Tenericutes* and *Fusobacteria* (Lima et al., 2017). Previous studies reported shared bacterial genera in colostrum and calf meconium (Klein-Jöbstl et al., 2019), feces collected a few days after birth (Zhu et al., 2021) or on d 14 (Hang et al., 2021), suggesting that bacteria in colostrum can be vertically transmitted to calves and may influence the development of calf gut microbiota. However, it is unclear which key bacterial species are transmitted and their dynamic changes in the gut microbiota of calves. Moreover, studies have shown that colostrum metabolites (Qi et al., 2018) are present in the neonatal calf gut, suggesting that they may contribute to early health and growth. However, it is largely unknown whether colostrum microbiota affects the colostrum metabolomic profile. Therefore, this study aims to explore the dynamic interconnection between colostrum and calf gut bacterial species and investigate the associations between colostrum microbiota and metabolites. We hypothesized that certain colostrum-derived bacterial species can persist in the calf gut, and their presence is associated with distinct metabolite profiles of colostrum.

This observational study included all heifers that conceived after natural breeding to an Angus bull (n = 25) and their Holstein-Angus crossbred offspring born between June to July 2023. All animals were from the same farm, and health was monitored before and throughout the entirety of the study. Each heifer or each calf was considered as an experimental unit. Before parturition, the Holstein heifers were housed together on pasture and subsequently transferred to a shared pen in the freestall barn that had access to the pasture as their estimated due dates approached. After parturition, the dams were transported to the milking parlor where each teat was cleaned, predipped with a germicidal iodine solution, stripped, and wiped off before colostrum collection. The colostrum was collected in a clean bucket followed by IgG measurement using a colostrometer, and 10 mL of the colostrum was aliquoted into a sterile 15-mL centrifuge tube and stored at −80°C for further analysis. Three low-quality colostrum samples containing <50 mg/mL of IgG were not fed to calves or used for further analysis. Each calf was fed 3 L of colostrum from either its dam or another dam within the same study group if the original dam's colostrum was of low quality. All calves were separated from their dams within the first 6 h after birth and housed individually in calf hutches, located on a freshly excavated pasture topped with fresh sand. From 2 to 14 d of age, the calves were fed 3 L of milk replacer (Land O'Lakes Cow's Match Jersey Blend) twice daily. From d 15 to 18, the calves gradually transitioned from milk replacer to whole milk, and from d 19 onward, they were fed whole milk. Starter grain was provided from d 15 onward. Fecal samples were aseptically collected from the recto-anal junction of individual calves on d 4, 7, 14, and 30 using sterile cotton swabs after cleaning the perianal area with 70% ethanol. All samples were immediately placed on ice, transported to the laboratory, and stored at −80°C until further processing.

To determine the absolute bacterial counts, the colostrum samples (n = 21) were plated after serial dilution on tryptic soy agar plates and incubated at 37°C for 18 h before counting. The colostrum and fecal microbiota were analyzed using the full-length 16S rRNA gene amplicon sequencing with the Oxford Nanopore platform (Oxford Nanopore Technologies, Oxford, UK) as described in our previous study (Rios de Alvarez et al., 2025). The taxonomic annotation was conducted on the filtered high-quality reads using the Emu pipeline and its default database (Curry et al., 2022). The α diversity assessed by Chao1 index and β diversity assessed by Bray–Curtis distance were analyzed using MicrobiomeAnalyst 2.0 (Lu et al., 2023). The Venn diagram displaying the shared bacterial species between colostrum and fecal samples collected at different time points was generated using the Bioinformatics and Evolutionary Genomics website (https://bioinformatics.psb.ugent.be/webtools/Venn/). To investigate whether colostrum microbiota affects colostrum metabolomics, a focal group (n = 9) of colostrum samples was selected based on microbiome differences across 3 clusters (3 samples per group) and sent to Creative Proteomics (New York, NY; https://www.creative-proteomics.com/) for ultra-performance liquid chromatography-MS analysis (Contreras-Correa et al., 2024). Metabolites were annotated with Compound Discoverer (v3.4) Software (Thermo Fisher) against mzCloud database and classified via PubChem. Metabolites identified in the negative and positive ion modes were combined, and for duplicates, the ion mode with the higher average across samples was retained in the dataset.

Statistical differences in plating counts were analyzed using a one-way ANOVA in R Studio (version 4.5.0). Differences in α diversity (Chao1 index) and β diversity (Bray–Curtis distance) of microbiota were analyzed using Kruskal–Wallis test and permutational multivariate ANOVA (**PERMANOVA**), respectively, in MicrobiomeAnalyst 2.0. Metabolomic pathways were identified and illustrated using MetaboAnalyst 6.0 (https://www.metaboanalyst.ca/; Huang et al., 2024). Differences in metabolomic profile evaluated by euclidean distance were analyzed using unsupervised principal component analysis (**PCA**) and PERMANOVA with vegan package in R Studio (version 4.5.0). Correlations between relative abundance of shared bacterial species (≥50% prevalence) in colostrum and calf feces, as well as between bacterial species and metabolites from the colostrum focal group, were analyzed using pairwise Spearman's rank correlations (R_Spearman_) with the Hmisc packing within RStudio (version 4.5.0). Statistical significance was set at *P* ≤ 0.05.

Colostrum samples yielded an average bacterial colony count of 4.31 × 10^6^ cfu/mL (6.6 log_10_ cfu/mL; SE = 2.64 × 10^6^) on tryptic soy agar plates (Figure 1A). One of the colostrum samples (Tag2025) with the lowest bacterial colony count failed 16S rRNA gene amplification and was not sequenced. An average of 107,125 classified sequencing reads were yielded for colostrum samples. After normalization, 430 different bacterial species were identified in the colostrum samples, and drastic individual variation in bacterial diversity (Figure 1B) and composition (Figure 1C) was observed across samples. *Lactococcus lactis* and *Megamonas rupellensis* were the only 2 species with a 100% prevalence in all 21 colostrum samples. *Streptococcus thermophilus*, *L. lactis*, and *Comamonas testosteroni* had the highest average relative abundances (16%, 15%, and 4%, respectively). Furthermore, colostrum samples differed in dominance by these 3 species, showing distinct bacterial compositions (*P*_Bray-Curtis_ = 0.001, Figure 1D), were grouped into 3 clusters. There were no differences in absolute bacterial counts (*P* = 0.16) between the 3 clusters. However, colostrum samples dominated by *C. testosteroni* showed higher bacterial richness, reflected by Chao1 index (*P* = 0.01), compared with those dominated by *S. thermophilus* and *L. lactis. Streptococcus thermophilus* and *L. lactis* are known probiotics that have been reported in raw cow milk and colostrum (Masoud et al., 2012; Yasir et al., 2024), whereas *C. testosteroni* is an environmental bacterium that is related to steroid degradation (Horinouchi et al., 2012). The *Comamonas* genus showed higher level in cow milk from farms with high subclinical mastitis incidence (Pang et al., 2018) and in the colon of calves fed untreated bulk tank milk (Deng et al., 2017), suggesting udder-microbiome dysbiosis of the cows that contained a high abundance of *Comamonas* and its risk to the health of newborn calves.

**Figure 1:**
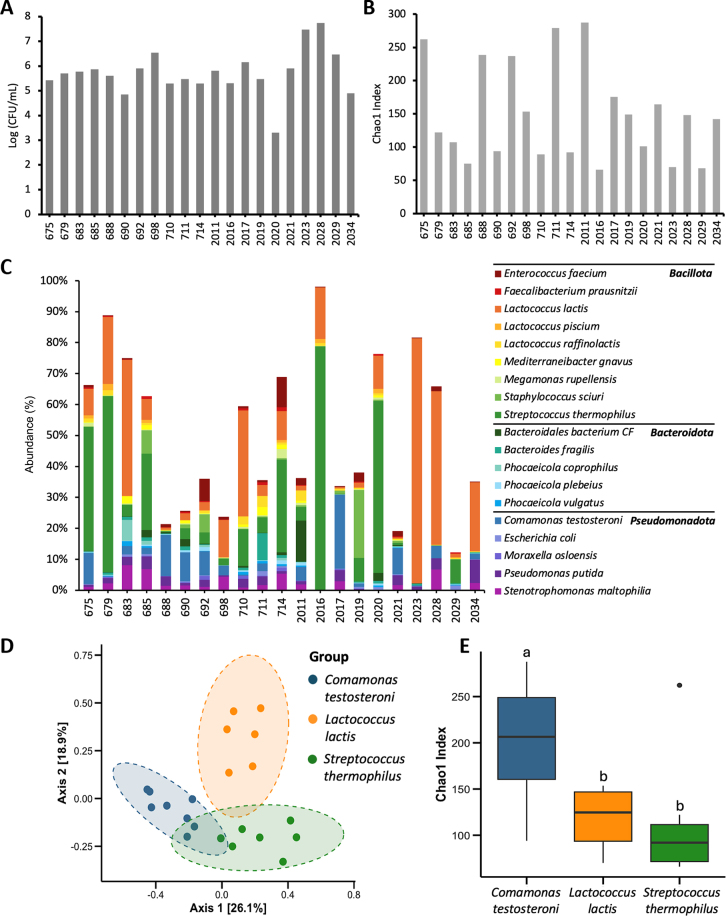
Dynamics of colostrum microbiota. (A) Bacterial counts (logarithm of colony forming units, log cfu/mL) in colostrum samples determined using tryptic soy agar (TSA) plates. (B) The Chao1 index. (C) Relative abundance of prevalent species (≥80% prevalence) in colostrum samples. (D) Principal coordinate analysis based on Bray–Curtis distances comparing microbiota composition among colostrum samples dominated by *Comamonas testosteroni*, *Lactococcus lactis*, or *Streptococcus thermophilus* (*P* = 0.001). The difference in Bray–Curtis distance was measured by the permutational multivariate ANOVA (PERMANOVA). (E) The Chao1 index of colostrum samples grouped by different dominant species (*P* = 0.017). Differing lowercase letters represent significant differences (*P* ≤ 0.05). The upper and lower edges of the box represent the interquartile range (25th and 75th percentiles), the center line indicates the median, the whiskers extend to 1.5 times the interquartile range, and the dots represent outliers.

We further investigated whether the prevalent and abundant colostrum bacterial species would dominate and persistently colonize in the neonatal calves. An average of 85,642 classified sequencing reads were generated for fecal samples. There were no species with 100% prevalence shared among colostrum and calf feces on d 4, 7, 14, and 30. However, we identified 15 shared species that were prevalent in more than 50% of colostrum samples and at each calf fecal sampling time point (Figure 2A), including *Escherichia coli* and *Mediterraneibacter gnavus*, which belong to genera recently reported to be shared between colostrum and calf feces for up to 2 mo of age (Urrutia-Angulo et al., 2025). The shared species made up approximately 20% of the colostrum bacterial composition, 67% of the fecal bacterial composition of d 4, 51% of d 7, 40% of d 14, and ultimately reduced to 15% of the fecal bacterial composition of d 30 (Figure 2B). *Streptococcus thermophilus* was the most abundant among the 15 shared species in colostrum but the abundance of this species was drastically less in calf feces. Conversely, the other 14 shared species, including *Bacteroides fragilis*, *E. coli*, and *M. gnavus*, accounted for a small portion of the colostrum microbiota but comprised 67% of the calf fecal microbiota on d 4. Additionally, colostrum and d 30 fecal microbiota each harbored a large number of unique species (Figure 2A), reflecting the distinct colonizing environments of colostrum and the calf gut. The reduced proportion of shared species and increased richness of the calf gut microbiota, further indicate diet- and age-related shifts in the microbial colonization niche (Amin and Seifert, 2021). Early colonization favors facultative anaerobes, such as *E. coli* and *Streptococcus* spp., and anaerobic simple-carbohydrate utilizers, including *B. fragilis* and *M. gnavus*, during the first 2 wk of life when calves are fed colostrum and milk replacer. The community subsequently transitions toward a more diverse assemblage of strict anaerobes, including fiber-fermenting taxa such as *Faecalibacterium prausnitzii*, following the introduction of whole milk and starter feed, which was also observed by Urrutia-Angulo et al. (2025). However, only 2 out of 15 shared species exhibited positive correlations in relative abundance between colostrum and calf fecal samples, particularly collected at d 30, including *Hungatella hathewayi* (R_Spearman_ = 0.42, *P* = 0.05) and *M. gnavus* (R_Spearman_ = 0.42, *P* = 0.05). Similarly, Hang et al. (2021) also detected minimal bacterial associations between colostrum and neonatal calf feces at birth or d 14. Nevertheless, our study identified a subset of bacterial species in colostrum capable of persisting in the calf gut during the first month of life. Although other environmental sources, such as milk replacer, starter grain, whole milk, or soil, may also harbor these species and contribute to their presence in the calf gut, their repeated detection in feces throughout the first month of life suggests the potential for early persistence. Colostrum could serve as one of the direct microbial reservoirs and early inocula for these species, influencing their colonization of the neonatal gut.

**Figure 2:**
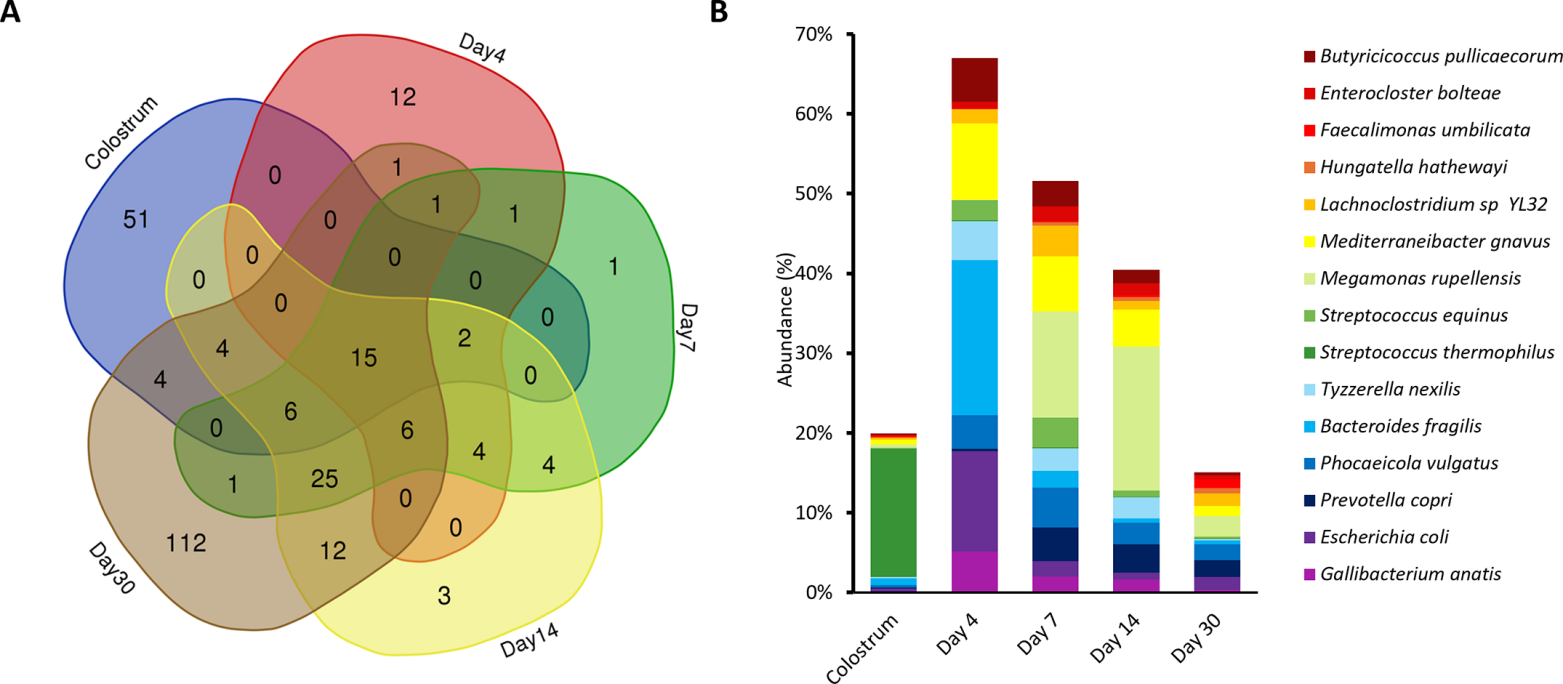
Unique and shared species in colostrum and calf fecal samples from d 4, 7, 14, and 30. (A) Venn diagram illustrating species with 50% prevalence unique to each day and shared species among the samples. (B) Relative abundance of species shared by colostrum and calf fecal samples with 50% prevalence.

Furthermore, the metabolomic analysis identified 405 metabolites present in colostrum samples, belonging to 7 different superclasses and 32 different subclasses. The superclass that included the most members were organic chemicals followed by lipids and heterocyclic compounds (Figure 3A), whereas the subclasses that contained the most members were fatty acids, carboxylic acids, and hydrocarbons. An unsupervised PCA was performed to illustrate the metabolic differences among 3 colostrum groups that exhibited distinct microbial composition (Figure 3B). The PERMANOVA revealed no significant differences (*P*_Euclidiean_ = 0.87) between the 3 groups, suggesting that colostrum microbiota was not the major driver of shaping the colostrum metabolomic profiles. However, we detected 1,379 significant Spearman correlations between bacterial species and metabolites in colostrum (*P* ≤ 0.05). We identified 56 strong positive (R_Spearman_ ≥ 0.85, *P* ≤ 0.05) and 98 strong negative (R_Spearman_ ≤ −0.85, *P* ≤ 0.05) correlations between colostrum bacterial species and metabolites (Figure 3C). Four metabolomic pathways, including phenylalanine metabolism; valine, leucine, and isoleucine biosynthesis; glycine, serine, and threonine metabolism; and starch and sucrose metabolism, were significantly enriched among metabolites positively correlated with colostrum bacteria (Figure 3D), whereas 5 pathways, including biosynthesis of UFA; valine, leucine, and isoleucine biosynthesis; one-carbon pool by folate; fatty acid biosynthesis; and porphyrin metabolism, were enriched among metabolites negatively correlated with colostrum bacteria (Figure 3E). These results suggest that colostrum-associated bacteria may preferentially support AA and carbohydrate metabolic activities while being inversely associated with lipid and cofactor biosynthetic processes, potentially shaping the nutritional and bioactive profile of colostrum available to the neonate.

**Figure 3:**
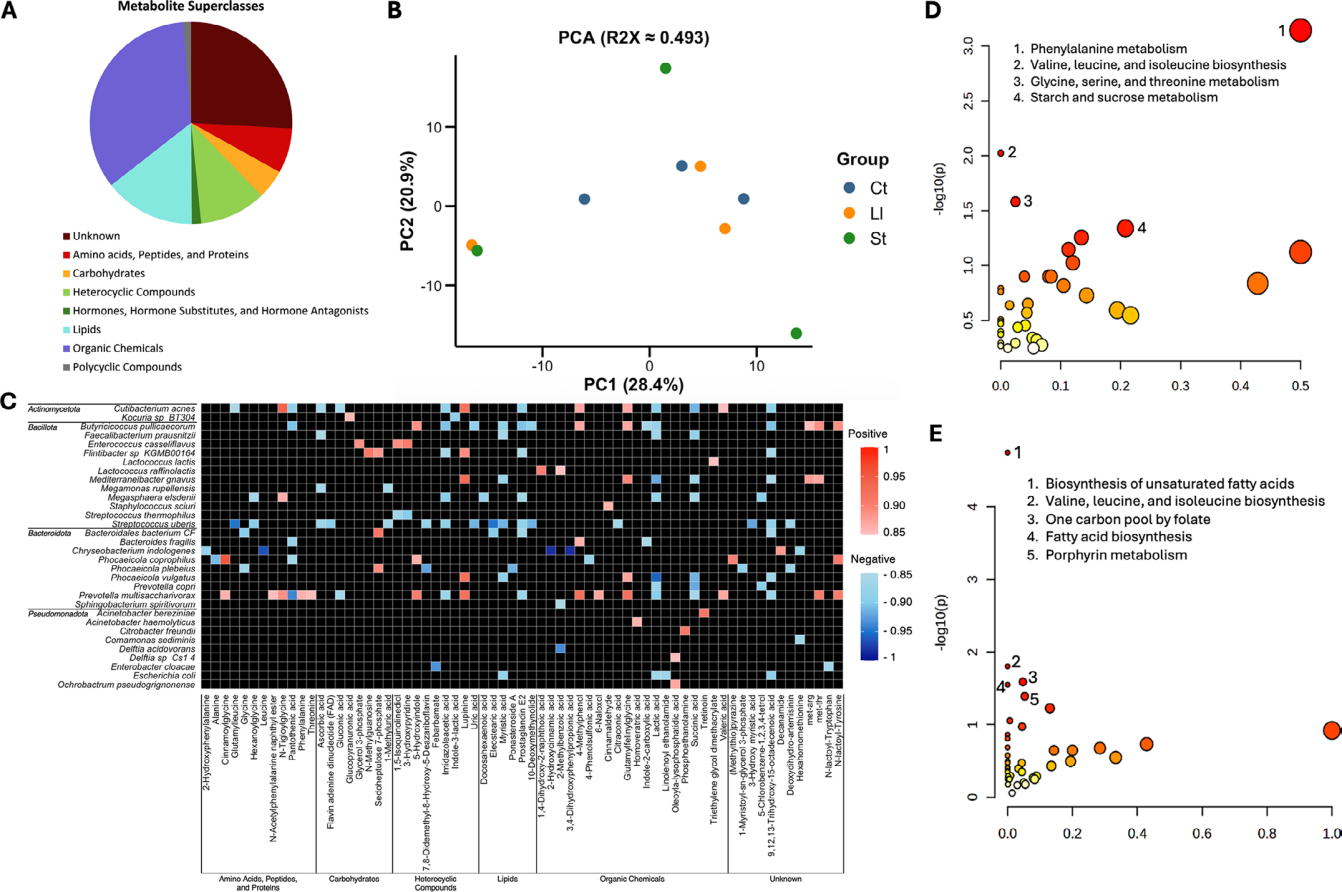
Colostrum metabolomic profile and its association with colostrum microbiota. (A) Distribution of metabolite superclasses in colostrum samples. (B) Principal component analysis (PCA) with the Euclidean distance comparing the differences in colostrum metabolome between colostrum samples dominated by different bacterial species (*P_vegan_* = 0.87). Ct = *Comamonas testosteroni*; Ll = *Lactococcus lactis*; St = *Streptococcus thermophilus*; PC1 = principal component 1; PC2 = principal component 2. (C) Heatmap illustrating strong Spearman correlations (R_Spearman_ ≥ 0.85 and R_Spearman_ ≤ −0.85, *P* ≤ 0.05) between bacterial species (≥50% prevalence) and metabolites in colostrum. Metabolomic pathway analysis of metabolites positively (D) or negatively (E) associated with colostrum bacterial species. The darker (redder) circles represent pathways with more significant enrichment, whereas circle size indicates the pathway impact score. Statistically significant, high-impact pathways are labeled.

Additionally, we identified several positive associations between bacterial species that were shared between colostrum and calf fecal samples (*B. fragilis*, *Butyricicoccus pullicaecorum*, *M. gnavus*, and *Phocaeicola vulgatus*) and certain microbial-derived metabolites (Figure 3C). *Butyricicoccus pullicaecorum*, a butyrate-producing gut probiotic (Eeckhaut et al., 2016), was positively correlated with 2 microbial-derived metabolites, 4-methylphenol (p-cresol) (R_Spearman_ = 0.87, *P* = 0.002) and 5-hydroxyindole (R_Spearman_ = 0.88, *P* = 0.001). 4-Methylphenol and 5-hydroxyindole are involved in the regulation and stimulation of intestinal motility, respectively (Toft et al., 2023; Waclawiková et al., 2023). 4-Methylphenol has also previously been identified as a naturally occurring metabolite in bovine milk, and was also positively associated with *B. fragilis* (R_Spearman_ = 0.86, *P* = 0.002), a prolific human milk oligosaccharide consumer that is an early colonizer of the human infant gut, yet its presence in bovine colostrum and ability to utilize bovine milk oligosaccharides remains uninvestigated (Buzun et al., 2024; Olm and Mueller, 2024). We also identified additional microbial-derived metabolites positively associated with other colostrum bacteria, including 1,4-dihydroxy-2-naphthoic acid, valeric acid (pentanoate), cinnamoylglycine, and threonine. 1,4-Dihydroxy-2-naphthoic acid, a precursor to vitamin K_2_, exhibits anti-inflammatory activity in the gut (Bentley and Meganathan, 1982) and stimulates ruminal bacterial growth and activity (Fenn et al., 2017; Chen et al., 2020). Interestingly, *Streptococcus uberis*, a pathogen associated with mastitis, exhibited the greatest number of strong negative correlations with metabolites (n = 16), such as the fatty acids (eleostearic acid [R_Spearman_ = −0.94, *P* < 0.001] and myristic acid [R_Spearman_ = −0.91, *P* < 0.001]), and the vitamins ascorbic acid (R_Spearman_ = −0.87, *P* = 0.002) and flavin adenine dinucleotide (**FAD**; R_Spearman_ = −0.89, *P* = 0.001). Ascorbic acid (vitamin C) is an essential antioxidant for newborn calves as they lack the ability to sufficiently produce adequate amounts and therefore must obtain it from the diet (Hidiroglou et al., 1995), and FAD is a crucial co-enzyme involved in energy metabolism and cell function (Hirano and Namihira, 2017). Many studies reported that mastitis alters the metabolomic profile in milk (Xi et al., 2017; Zhu et al., 2024), suggesting that the presence of mastitis-causing pathogens in colostrum may contribute to nutrient degradation and restrict essential metabolites delivered to calves. These findings indicate that while the bovine colostrum microbiota does not dramatically alter the overall metabolomic profile of colostrum, specific bacterial species may produce unique microbial-derived metabolites and degrade certain critical nutrients that may further influence colostrum quality and calf development.

Certain limitations of this study need to be acknowledged. We primarily identified colostrum bacterial species with the potential to colonize and persist in the calf gut using Venn analyses and Spearman correlations between colostrum microbiota and fecal microbiota collected from d 4 until d 30. Including a negative control group without colostrum feeding, would provide valuable insights into the dynamics (e.g., presence or absence, and relative abundance) of colostrum-associated bacteria before and immediately after birth, or with and without colostrum intake. Such data would enable a more rigorous assessment of the potential causal effects of colostrum microbiota on calf gut development. Additionally, the sample size for the metabolomic analysis was relatively small, and a larger sample size would increase our statistical power.

In conclusion, this present study provides a deeper understanding of the colostrum microbiome, particularly its relationship with calf gut microbiota at the species level and with the colostrum metabolomic profile. We identified 15 bacterial species in colostrum that were persistently detected in calf feces throughout the first month of life, in which 4 of these (*B. fragilis*, *B. pullicaecorum*, *M. gnavus*, and *P. vulgatus*) showed strong positive associations with colostrum metabolites. Further investigations using direct oral supplementation of these bacteria or metabolites to calves are warranted to better elucidate their roles in calf microbiota development, growth, and health. Moreover, the strong associations observed between specific colostrum bacterial species and metabolites suggest potential metabolic functions of these bacteria that may influence colostrum quality. These larger scoped hypotheses should be further validated using an integrated approach combining culture-based methods with multiomics analyses (e.g., metabolomics and transcriptomics).
